# Effect of Abdominal Binding on Diaphragmatic Neuromuscular Efficiency, Exertional Breathlessness, and Exercise Endurance in Chronic Obstructive Pulmonary Disease

**DOI:** 10.3389/fphys.2018.01618

**Published:** 2018-11-14

**Authors:** Sara J. Abdallah, Benjamin M. Smith, Courtney Wilkinson-Maitland, Pei Zhi Li, Jean Bourbeau, Dennis Jensen

**Affiliations:** ^1^Clinical Exercise & Respiratory Physiology Laboratory, Department of Kinesiology and Physical Education, McGill University, Montreal, QC, Canada; ^2^Respiratory Epidemiology & Clinical Research Unit, Division of Respiratory Medicine, Department of Medicine, Research Institute of the McGill University Health Centre, McGill University, Montreal, QC, Canada; ^3^McGill Research Centre for Physical Activity and Health, McGill University, Montreal, QC, Canada

**Keywords:** breathlessness, abdominal binding, diaphragm, neuromuscular efficiency, exercise endurance

## Abstract

We tested the hypothesis that abdominal binding (AB) would reduce breathlessness and improve exercise tolerance by enhancing neuromuscular efficiency of the diaphragm during exercise in adults with chronic obstructive pulmonary disease (COPD). In a randomized, controlled, crossover trial, 20 adults with COPD (mean ± SD FEV_1_, 60 ± 16% predicted) completed a symptom-limited constant-load cycle endurance exercise test at 75% of their peak incremental power output with concomitant measures of the diaphragm electromyogram (EMGdi) and respiratory pressures without (CTRL) vs. with AB sufficient to increase end-expiratory gastric pressure (Pga,ee) by 6.7 ± 0.3 cmH_2_O at rest. Compared to CTRL, AB enhanced diaphragmatic neuromuscular efficiency during exercise (*p* < 0.05), as evidenced by a 25% increase in the quotient of EMGdi to tidal transdiaphragmatic pressure swing. By contrast, AB had no demonstrable effect on exertional breathlessness and exercise tolerance; spirometry and plethysmography-derived pulmonary function test parameters at rest; and cardiac, metabolic, breathing pattern, inspiratory reserve volume and EMGdi responses during exercise (all *p* > 0.05 vs. CTRL). In conclusion, enhanced neuromuscular efficiency of the diaphragm during exercise with AB was not associated with relief of exertional breathlessness and improved exercise tolerance in adults with COPD.

**Clinical Trial Registration:** ClinicalTrials.gov Identifier: NCT01852006.

## Introduction

In people with chronic obstructive pulmonary disease (COPD), lung hyperinflation shortens the length of the diaphragm, thereby compromising its length-tension relationship and area of apposition to the rib cage (Cassart et al., 1997; Laghi and Tobin, 2003). Collectively, these changes promote diaphragmatic neuromuscular inefficiency by decreasing diaphragm pressure-generating capacity and provoking high levels of diaphragm electrical activation (EMGdi), particularly during exercise when dynamic lung hyperinflation further shortens and weakens the diaphragm (Sinderby et al., 2001; Finucane and Singh, 2012). Diaphragmatic neuromuscular inefficiency has been mechanistically linked to abnormally high levels of exertional breathlessness and abnormally low levels of exercise tolerance in COPD (Laghi et al., 1998, 2004). It follows that any intervention capable of enhancing diaphragmatic neuromuscular efficiency may decrease exertional breathlessness and improve exercise tolerance in adults with COPD. Indeed, Laghi et al. (1998) reported that improvements in diaphragmatic neuromechanical coupling after lung volume reduction surgery (LVRS) in patients with severe emphysema correlated with relief of breathlessness at rest and improved 6-min walking distance.

In 1934, Alexander and Kontz (1934) and Gordon (1934) reported symptomatic improvement of breathlessness following application of a belt around the abdomen in adults with various pulmonary diseases, including bronchitis, emphysema, and asthma. In keeping with these observations, Celli et al. (1985) reported that abdominal binding (AB) sufficient to increase end-expiratory gastric pressure (Pga,ee) by 8 cmH_2_O increased maximal voluntary transdiaphragmatic pressure-generating capacity by 13 cmH_2_O (93%), decreased the perception of breathlessness at rest, and increased exercise tolerance in a symptomatic patient with severe COPD and a large midline hernia of the anterior abdominal wall. Presumably, this improvement in diaphragm pressure-generating capacity via AB reflected the combination of reduced abdominal wall compliance, increased intra-abdominal pressure, improved operating length of the diaphragm due to its ascent to a more mechanically advantageous (cephalad) position, increased area of diaphragm apposition to the rib cage, and increased diaphragm-rib cage insertional forces (Koo et al., 2015).

Recently, West et al. (2012) reported improvements in static lung volumes and capacities following AB in people with cervical spinal cord injury (SCI) as well as in healthy adults. For example, AB decreased functional residual capacity by 0.75 l (23%) in SCI and 0.46 l (14%) in health; increased inspiratory capacity (IC) by 0.47 l in SCI (20%) and 0.33 l (11%) in health; and increased inspiratory reserve volume (IRV) by 0.49 l (29%) in SCI and 0.38 l (16%) in health. A subsequent study by West et al. (2014) demonstrated that, compared to the unbound condition, AB shifted tidal breathing to lower and more mechanically advantageous end-expiratory and end-inspiratory lung volumes during submaximal exercise in athletes with cervical SCI.

We recently demonstrated that increasing Pga,ee by 6.6 ± 0.6 cmH_2_O (mean ± SE) at rest via AB markedly improved diaphragmatic neuromuscular efficiency – quantified as the quotient of tidal transdiaphragmatic pressure swing (Pdi,tidal) to the root mean square of the crural diaphragm electromyogram (EMGdi,rms) – by 85–90% during cycle endurance exercise testing in healthy young men (Abdallah et al., 2017). Despite this improvement, AB had no effect on exertional breathlessness and exercise endurance, likely because diaphragmatic neuromuscular inefficiency is not the proximate source of exertional breathlessness and exercise limitation in healthy young adults (Abdallah et al., 2017). Nevertheless, the collective results of Alexander and Kontz (1934), Gordon (1934), Celli et al. (1985), West et al. (2012, 2014) and ourselves (Abdallah et al., 2017) provide a physiological rationale for the use of AB as a potentially effective non-pharmacological means of alleviating exertional breathlessness and improving exercise tolerance in adults with COPD by improving dynamic operating lung volumes and/or enhancing diaphragmatic neuromuscular efficiency.

Therefore, the primary aim of this study was to evaluate the effect of AB on the inter-relationships between diaphragmatic neuromuscular efficiency, exertional breathlessness and exercise endurance in adults with COPD.

## Materials and Methods

### Study Design

This single-center, randomized, controlled, crossover trial was conducted at the McGill University Health Centre in Montreal, QC, Canada (Clinicaltrials.gov identifier: NCT01852006). The study protocol and informed consent form received ethics approval from the Research Institute of the McGill University Health Centre (13-075-BMA) in accordance with the *Declaration of Helsinki*.

After providing written and informed consent, participants completed a screening/familiarization visit followed by two intervention visits randomized to order. All visits were separated by ≥48-h. *Visit 1* included: medical history and clinical assessment; evaluation of activity-related breathlessness using the modified Medical Research Council dyspnoea scale (Bestall et al., 1999), the Baseline Dyspnea Index (Mahler et al., 1984) and the Oxygen Cost Diagram (McGavin et al., 1978); evaluation of health status using the COPD Assessment Test (Jones et al., 2009); evaluation of anxiety and depression using the Hospital Anxiety and Depression scale (Zigmond and Snaith, 1983); post-bronchodilator (400 μg salbutamol) spirometry; and a symptom-limited incremental cardiopulmonary cycle exercise test (CPET) to determine peak power output (PPO), defined as the highest power output that the participant was able to sustain for ≥30-s. During *Visits 2 and 3*, participants first inhaled 400 μg of salbutamol. The gastro-esophageal electrode-balloon catheter used to record EMGdi,rms and respiratory pressures (*see below*) was then inserted and positioned in accordance with established techniques (Jensen et al., 2011). During the AB visit, the abdominal binder was applied and optimally fitted (*see below*). Once the AB was optimally fitted, the gastro-esophageal electrode-balloon catheter was re-positioned to achieve optimal recordings of EMGdi during resting breathing (i.e., positioned such that the amplitude of EMGdi during inspiration was greatest in electrode pairs 1 and 5, and lowest in electrode pair 3) (Jensen et al., 2011). In this way, the recording electrodes were similarly positioned at the diaphragm’s electrically active center under both CTRL and AB conditions. Thereafter, participants completed spirometry and plethysmography followed by a symptom-limited constant-load cycle CPET at 75% PPO. Participants were permitted to use their respiratory medications according to their regular schedule. Participants were randomized in a 1:1 ratio according to a computer-generated block randomization schedule (Block size = 4) prepared by a third-party statistician not involved in the trial.

### Participants

Participants were recruited from the Montreal Chest institute of the McGill University Health Centre, and included men and women aged ≥40 y with Global Initiative for Obstructive Lung Disease (GOLD) stage II or III COPD (Vogelmeier et al., 2017), cigarette smoking history ≥15 pack-years, and no change in medication dosage or frequency of administration with no exacerbation(s) and/or hospitalization(s) in the preceding 6-weeks. Exclusion criteria were: presence of medical conditions other than COPD that could contribute to breathlessness and/or exercise intolerance; use of domiciliary oxygen; exercise-induced oxyhemoglobin desaturation to <80% on room air; and body mass index <18.5 or ≥35.0 kg/m^2^.

### Intervention

A commercially available binder (493R Universal Back Support; McDavid Inc., Woodridge, IL, United States) that has been described in detail elsewhere (West et al., 2012) was used to bind the abdomen. The binder was fitted with the upper edge below the costal margin so that it interfered minimally with rib-cage movement. The desired degree of abdominal compression – defined as a 5–8 cmH_2_O increase in Pga,ee – was achieved by tightening Velcro fasteners at the front of the binder with participants breathing normally while seated at rest. We recently demonstrated that this level of abdominal compression enhanced diaphragmatic neuromuscular efficiency during exercise in healthy young men, as evidenced by an 85–90% increase in the quotient of Pdi,tidal to EMGdi,rms (Abdallah et al., 2017). Furthermore, West et al. (2012) demonstrated that this level of abdominal compression was associated with significantly greater improvements in diaphragm function than increasing Pga,ee by 1.0–3.5 cmH_2_O in healthy adults and people with cervical SCI.

### Procedures

#### Pulmonary Function Testing

Spirometry and plethysmography were performed with participants seated using automated equipment (Vmax Encore^TM^ 29C, CareFusion, Yorba Linda, CA, United States; Medisoft Body Box 5500^®^, Medisoft Belgium, Sorinnes, Belgium) and according to recommended techniques (Macintyre et al., 2005; Miller et al., 2005a,b; Wanger et al., 2005). Measurements were referenced to predicted normal values (Briscoe and Dubois, 1958; Burrows et al., 1961; Crapo et al., 1981; Hankinson et al., 1999).

#### Cardiopulmonary Exercise Testing

Exercise tests were conducted on an electronically braked cycle ergometer (Lode Corival, Lode BV Medical Technology, Groningen, Netherlands) using a computerized CPET system (Vmax Encore^TM^ 29C). Incremental CPETs consisted of a baseline rest period of ≥6-min, followed by 10 W/min increases in power output to symptom-limitation. Constant-load CPETs consisted of a baseline rest period of ≥6-min, followed by 1-min of unloaded pedaling and then a step increase in power output to 75% PPO maintained to symptom-limitation. Cardiac, metabolic, gas exchange, and breathing pattern parameters were collected breath-by-breath and analyzed as previously described (Abdallah et al., 2017). Inspiratory capacity maneuvers were performed at rest, every 2-min during CPET, and at end-exercise (Guenette et al., 2013). Measurements of PPO, peak oxygen uptake and peak heart rate were referenced to the predicted normal values of Jones et al. (1985).

Published methods were used to analyze breath-by-breath measures of EMGdi,rms and of esophageal (Pes), gastric (Pga) and transdiaphragmatic pressure (Pdi = Pga-Pes) recorded from a gastro-esophageal electrode-balloon catheter (Guangzhou Yinghui Medical Equipment Ltd., Guangzhou, China) (Jensen et al., 2011; Mendonca et al., 2014; Abdallah et al., 2017). Maximum voluntary EMGdi,rms was identified as the largest of all EMGdi,rms values obtained from IC maneuvers performed either at rest or during exercise. Tidal swings in Pes (Pes,tidal), Pga (Pga,tidal), and Pdi (Pdi,tidal) were calculated as the difference between peak tidal inspiratory and peak tidal expiratory Pes, Pga, and Pdi, respectively. The quotient of Pdi,tidal to EMGdi,rms was used as an index of diaphragmatic neuromuscular efficiency (Abdallah et al., 2017).

Using Borg’s modified 0–10 category ratio scale (Borg, 1982), participants rated the intensity and unpleasantness of their breathlessness, as well as the intensity of their leg discomfort at rest, every 2-min during CPET, and at end-exercise. At end-exercise, participants were asked to identify their locus of symptom limitation (breathlessness, leg discomfort, combination of breathlessness, and leg discomfort, other); to quantify the percentage contribution of their selection to exercise cessation; and identify qualitative phrases that best described their breathlessness at end-exercise (O’Donnell et al., 2000).

### Outcomes

#### Primary Outcomes

The primary outcome was the difference in breathlessness intensity ratings during exercise at isotime under AB vs. CTRL conditions, where isotime was defined as the highest equivalent 2-min interval of exercise completed by a given participant during each of the constant-load CPETs. The co-primary outcome was the difference in exercise endurance time (EET) under AB vs. CTRL conditions, where EET was defined as the duration of loaded pedaling during constant-load CPET.

#### Secondary Outcomes

Spirometry and plethysmography-derived pulmonary function test parameters; physiological and perceptual parameters measured at rest, at standardized submaximal times during constant-load CPETs, and at peak-exercise (defined as the average of the last 30-s of loaded pedaling); reasons for stopping exercise; percentage contribution of breathlessness and leg discomfort to exercise cessation; and qualitative descriptors of breathlessness at end-exercise.

### Statistical Methods

Using a two-tailed paired subject formula with α = 0.05, β = 0.90 and an expected effect size of 0.80 (Faul et al., 2009), we estimated that at least 19 participants were needed to detect a minimal clinically important difference of ±1 Borg unit in breathlessness intensity ratings (Ries, 2005) at isotime and of ±101-s in EET (Puente-Maestu et al., 2009) under AB vs. CTRL conditions.

Participants who completed both AB and CTRL arms of the trial were included in the analysis. Linear mixed-model regression with random intercepts was used to analyze differences in EET as well as in all physiological and perceptual responses to constant-load CPET under AB and CTRL conditions. Two-tailed paired *t*-tests were used to compare the effects of AB vs. CTRL on: spirometry and plethysmography-derived pulmonary function test parameters; maximal voluntary EMGdi,rms; and the percentage contribution of breathlessness and leg discomfort to exercise cessation. Fisher’s exact test was used to compare the effect of AB vs. CTRL on the selection frequencies of reasons for stopping exercise as well as the descriptors of breathlessness at end-exercise. Data were analyzed using SAS statistical package, version 9.4 (SAS Institute Inc., Cary, NC, United States) and SigmaStat, version 3.5 (Systat Software Inc., San Jose, CA, United States). Statistical significance was set at *p* < 0.05 and values are reported as mean ± SEM unless stated otherwise.

## Results

**FIGURE 1:**
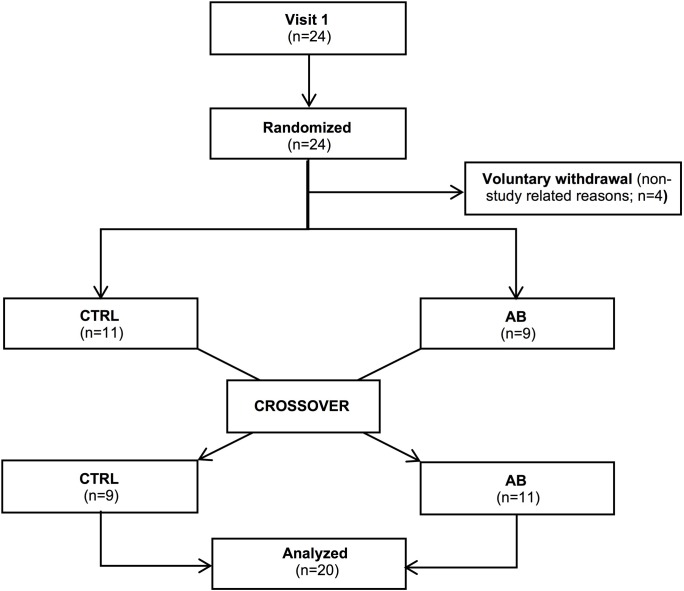
Consort diagram of the study population.

Twenty-four participants were randomized into the trial. Four of these participants dropped out during follow-up for non-study related reasons (Figure 1). Baseline characteristics of the 20 participants (13 men) who completed the trial are presented in Table 1. By design, AB increased Pga,ee by 6.7 ± 0.3 cmH_2_O above its baseline value during the AB visit.

**Table 1:** Baseline participant characteristics (*n* = 20).

Parameter	Value
Male:Female, *n*	13:7
Age, years	69.8 ± 8.7
Height, cm	170.1 ± 9.9
Body mass, kg	78.4 ± 15.5
Body mass index, kg⋅m^−2^	27.1 ± 1.1
Smoking history, pack-years	56.1 ± 30.1
**Post-bronchodilator spirometry**	
FEV_1_, L (% predicted)	1.56 ± 0.57 (60 ± 16)
FEV_1_/FVC, %	46.3 ± 12.3
FEF_25–75%_, L⋅s^−1^ (% predicted)	0.57 ± 0.31 (23 ± 11)
PEF, L⋅s^−1^ (% predicted)	4.55 ± 1.98 (59 ± 18)
**Breathlessness and health status**	
mMRC score, 0–4	1.8 ± 0.9
BDI focal score, out of 12	6.0 ± 2.0
Oxygen cost diagram, % full scale	51 ± 12
CAT score, out of 40	17.0 ± 7.8
HADS score, out of 42	9.8 ± 4.9

*Values are mean ± SD. FEV_1_, forced expiratory volume in 1-s; FEV_1_/FVC, FEV_1_ to forced vital capacity ratio; FEF_25–75%_, forced expiratory flow between 25 and 75% of the FVC maneuver; PEF, peak expiratory flow; mMRC, modified Medical Research Council dyspnoea scale; BDI, Baseline Dyspnoea Index; CAT, COPD Assessment Test; HADS, Hospital Anxiety and Depression Scale.*

### Primary Outcomes

**FIGURE 2:**
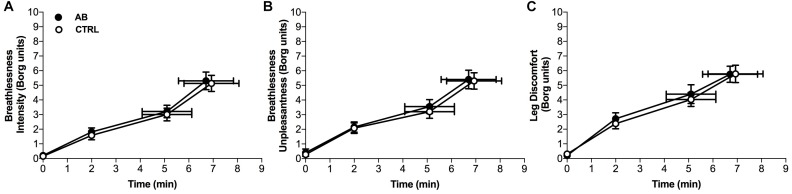
Effects of abdominal binding (AB) vs. control (CTRL) on **(A)** breathlessness intensity, **(B)** breathlessness unpleasantness and **(C)** leg discomfort during constant-load cycle endurance exercise testing at 75% of peak incremental power output in adults with chronic obstructive pulmonary disease (*n* = 20). Data points are mean ± SEM values at rest, at standardized submaximal times during exercise (including isotime of 5.1 ± 1.0 min), and at peak exercise.

Compared to CTRL, AB had no effect on breathlessness intensity ratings at isotime (AB, 3.2 ± 0.4 Borg units vs. CTRL, 3.0 ± 0.4 Borg units; *p* = 0.454) or on EET (AB, 6.7 ± 1.1 min vs. CTRL, 6.9 ± 1.1 min; *p* = 0.853) (Figure 2). To assess for a possible confounding order effect on our primary outcomes, breathlessness intensity ratings at isotime and EET were compared between *Visits 2 and 3.* There was no statistically significant effect of visit order on breathlessness intensity ratings at isotime (*Visit 2*, 3.2 ± 0.5 Borg units vs. *Visit 3*, 3.1 ± 0.4 Borg units; *p* = 0.873) or on EET (*Visit 2*, 7.3 ± 1.3 min vs. *Visit 3*, 6.4 ± 1.0 min; *p* = 0.079).

### Secondary Outcomes

#### Pulmonary Function

Compared to CTRL, AB had no effect on spirometry and plethysmography-derived pulmonary function test parameters at rest (Table 2).

**Table 2:** Effect of abdominal binding (AB) on spirometry and plethysmography-derived pulmonary function test parameters in adults with chronic obstructive pulmonary disease (*n* = 20).

Parameter	Control	AB
FEV_1_, L	1.53 ± 0.56	1.54 ± 0.64
FEV_1_/FVC, %	45.9 ± 12.7	45.1 ± 13.1
FEF_25–75%_, L⋅s^−1^	0.56 ± 0.29	0.52 ± 0.24
PEF, L⋅s^−1^	4.23 ± 1.63	4.16 ± 1.57
TLC, L (% predicted)	7.14 ± 1.39 (117 ± 19)	6.89 ± 1.45
RV, L (% predicted)	3.37 ± 0.88 (150 ± 45)	3.35 ± 0.99
FRC, L (% predicted)	4.61 ± 1.12 (140 ± 32)	4.28 ± 1.12
IC, L (% predicted)	2.55 ± 0.70 (89 ± 15)	2.67 ± 0.75
sRaw, cmH_2_O⋅L⋅s^−1^ (% predicted)	17.1 ± 11.2 (406 ± 261)	20.6 ± 15.5

*Values are mean ± SD. FEV_1_, forced expiratory volume in 1-s; FEV_1_/FVC, FEV_1_ to forced vital capacity ratio; FEF_25–75%_, forced expiratory flow between 25 and 75% of the FVC maneuver; PEF, peak expiratory flow; TLC, total lung capacity; RV, residual volume; FRC, functional residual capacity; IC, inspiratory capacity; sRaw, specific airway resistance.*

#### Physiological and Perceptual Responses to Exercise

**FIGURE 3:**
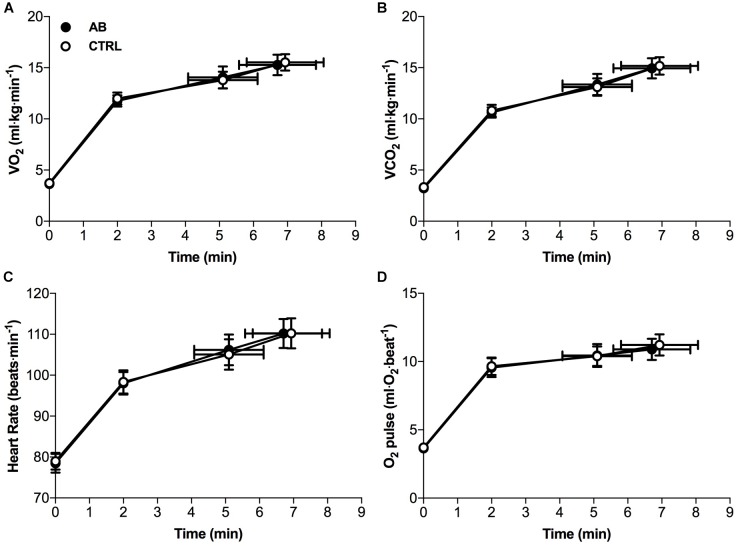
Effects of abdominal binding (AB) vs. control (CTRL) on **(A)** the rate of oxygen consumption (VO_2_), **(B)** the rate of carbon dioxide production (VCO_2_), **(C)** heart rate and **(D)** oxygen pulse (O_2_ pulse) during constant-load cycle endurance exercise testing at 75% of peak incremental power output in adults with chronic obstructive pulmonary disease (*n* = 20). Data points are mean ± SEM values at rest, at standardized submaximal times during exercise (including isotime of 5.1 ± 1.0 min), and at peak exercise.

**FIGURE 4:**
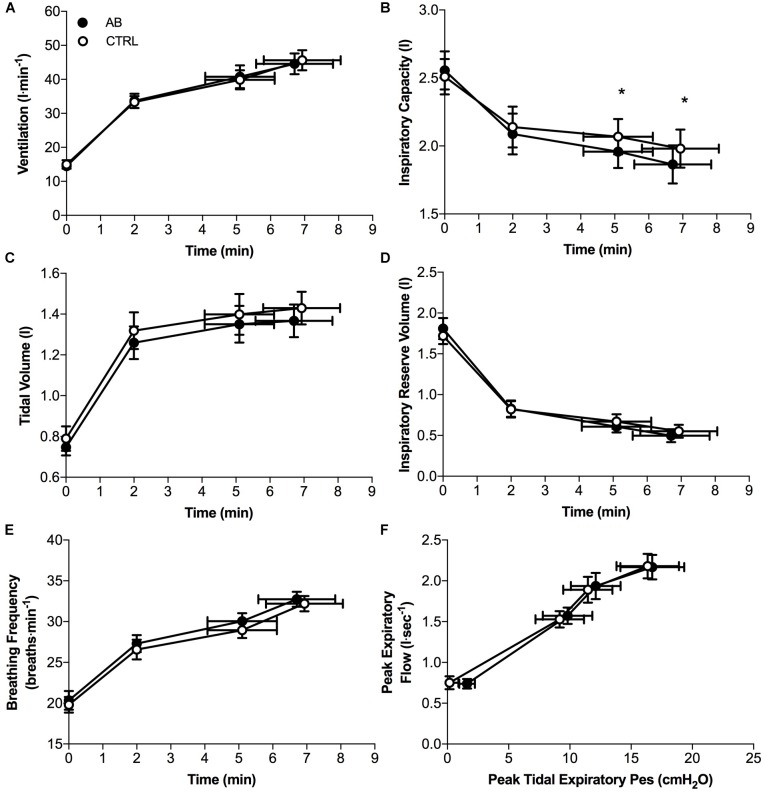
Effects of abdominal binding (AB) vs. control (CTRL) on **(A)** ventilation, **(B)** inspiratory capacity, **(C)** tidal volume, **(D)** inspiratory reserve volume, **(E)** breathing frequency, and **(F)** peak expiratory flow during constant-load cycle endurance exercise testing at 75% of peak incremental power output in adults with chronic obstructive pulmonary disease (*n* = 20). Pes, esophageal pressure. Data points are mean ± SEM values at rest, at standardized submaximal times during exercise (including isotime of 5.1 ± 1.0 min), and at peak exercise. ^∗^*p* < 0.05 vs. CTRL.

Except for small and isolated decreases in IC at isotime (AB, 1.96 ± 0.12 l vs. CTRL, 2.07 ± 0.13 l; *p* = 0.043) and at peak exercise (AB, 1.86 ± 0.14 l vs. CTRL, 1.98 ± 0.14 l; *p* = 0.024), AB had no demonstrable effect on cardiac, metabolic, ventilatory, breathing pattern and IRV parameters at rest or during exercise (Figures 3, 4).

Compared to CTRL, AB had no significant effect on maximal voluntary EMGdi,rms (AB, 162 ± 10 μV vs. CTRL, 160 ± 10 μV; *p* = 0.737). Peak inspiratory Pes values recorded during serial IC maneuvers did not change significantly from rest (AB, −24.6 ± 2.1 cmH_2_O vs. CTRL, −24.9 ± 1.5 cmH_2_O; *p* = 0.847) and throughout exercise (e.g., AB, −23.1 ± 1.4 cmH_2_O vs. CTRL, −22.8 ± 1.8 cmH_2_O at end-exercise; *p* = 0.816). Peak inspiratory Pdi values recorded during serial IC maneuvers performed at rest and throughout exercise were significantly increased by 4.4–8.3 cmH_2_O (10–22%) under AB vs. CTRL conditions [e.g., AB, 50.9 ± 2.2 cmH_2_O vs. CTRL, 46.4 ± 2.5 cmH_2_O at rest (*p* = 0.014); and AB, 46.5 ± 2.2 cmH_2_O vs. CTRL, 38.3 ± 2.2 cmH_2_O at end-exercise (*p* = 0.001)].

**FIGURE 5:**
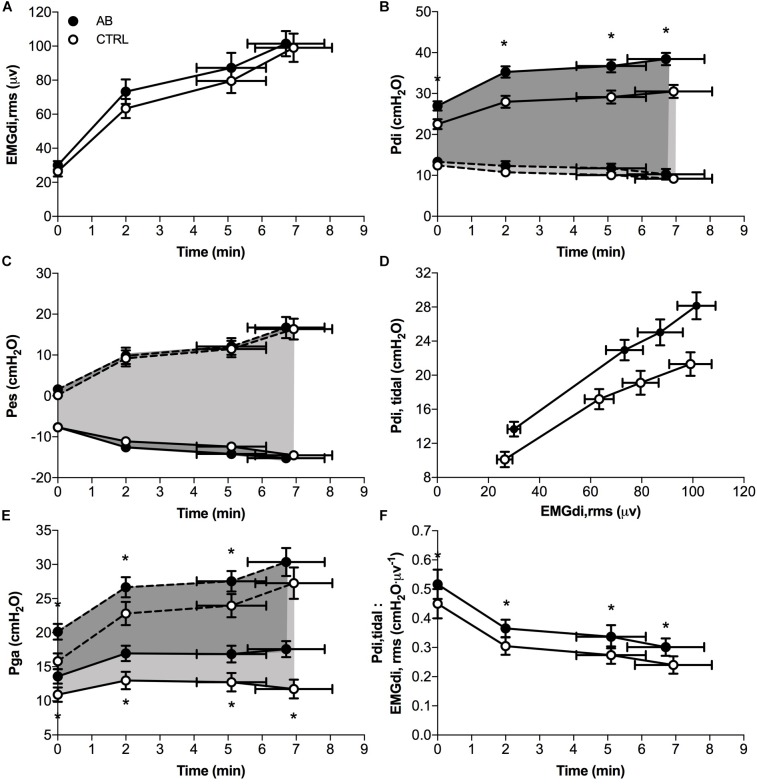
Effects of abdominal binding (AB) vs. control (CTRL) on **(A)** the root mean square of the crural diaphragm electromyogram (EMGdi,rms), **(B)** transdiaphragmatic pressure (Pdi), **(C)** esophageal pressure (Pes), **(D)** tidal Pdi swing (Pdi,tidal) vs. EMGdi,rms, **(E)** gastric pressure (Pga) and **(F)** the quotient of Pdi,tidal to EMGdi,rms (an index of diaphragmatic neuromuscular efficiency) responses during constant-load cycle endurance exercise testing at 75% of peak incremental power output in adults with chronic obstructive pulmonary disease (*n* = 20). Dashed lines in panels **B,C,E** denote peak tidal expiratory Pdi, Pes, and Pga, respectively. Data points are mean ± SEM values at rest, at standardized submaximal times during exercise (including isotime of 5.1 ± 1.0 min), and at peak exercise. ^∗^*p* < 0.05 vs. CTRL.

EMGdi,rms (Figure 5A) and Pes (Figure 5C) responses during exercise were significantly different under AB vs. CTRL conditions. Peak tidal inspiratory Pga and peak tidal expiratory Pga were consistently higher at rest and during exercise with vs. without AB (Figure 5E). Similarly, peak tidal inspiratory Pdi and Pdi,tidal were significantly higher at rest and during exercise under AB vs. CTRL conditions (Figure 5B). Finally, enhanced neuromuscular efficiency of the diaphragm with vs. without AB was evidenced by the consistently higher Pdi,tidal at any given EMGdi,rms during exercise (Figure 5D). Indeed, the quotient of Pdi,tidal to EMGdi,rms increased by an average of ∼25% at each measurement time during exercise under AB vs. CTRL conditions (Figure 5F).

Compared to CTRL, AB had no effect on the locus of symptom-limitation (Breathlessness: AB, *n* = 7 vs. CTR, *n* = 6; Leg discomfort: AB, *n* = 6 vs. CTRL, *n* = 6; and Combination of breathlessness and leg discomfort: AB, *n* = 7 vs. CTRL, *n* = 7). The relative contributions of breathlessness (AB, 44 ± 7% vs. CTRL, 47 ± 8%; *p* = 0.731) and leg discomfort (AB, 48 ± 7% vs. CTRL, 52 ± 8%; *p* = 0.531) to exercise cessation were not different under AB vs. CTRL conditions. Similarly, the selection frequency of breathlessness descriptors at end-exercise was not significantly different in AB vs. CTRL (data not shown).

## Discussion

The main finding of this randomized controlled trial was that AB enhanced neuromuscular efficiency of the diaphragm during exercise but had no effect on exertional breathlessness and exercise endurance in adults with COPD.

In keeping with the results of earlier studies in health (Hussain et al., 1985; Abdallah et al., 2017), SCI (West et al., 2012, 2014) and COPD (Alexander and Kontz, 1934; Gordon, 1934; Celli et al., 1985; Dodd et al., 1985), AB significantly enhanced pressure-generating capacity of the diaphragm at rest and throughout exercise. Presumably, by increasing intra-abdominal pressure, AB functionally “strengthened” the diaphragm and enhanced its pressure-generating capacity by improving its length-tension relationship, thus enabling the diaphragm to initiate its inspiratory contraction at a more favorable length (Koo et al., 2015). Furthermore, cephalad displacement of the diaphragm with AB likely increased the area of diaphragmatic apposition to the rib cage with attendant increases in the inflationary action of the diaphragm on the lower rib cage (Koo et al., 2015). AB presumably also minimized caudal shift of the diaphragm by reducing abdominal wall compliance, thus decreasing the velocity of diaphragm shortening (Koo et al., 2015). Collectively, these mechanically advantageous adaptations are likely responsible for the ∼25% improvement in diaphragmatic neuromuscular efficiency during exercise with vs. without AB in adults with COPD.

Despite enhanced diaphragmatic neuromuscular efficiency, AB had no effect on exertional breathlessness and EET. This is in contrast to the results of LVRS studies in COPD, wherein enhanced diaphragmatic neuromuscular efficiency correlated with relief of exertional breathlessness and increased exercise capacity (Laghi et al., 1998, 2004; Lahrmann et al., 1999; Gorman et al., 2005). Enhanced diaphragmatic neuromuscular efficiency following LVRS is secondary to enhanced respiratory mechanics, as evidenced by reduced static and dynamic lung hyperinflation and improved breathing pattern (Laghi et al., 1998, 2004; Lahrmann et al., 1999; Gorman et al., 2005). By increasing the area of diaphragmatic apposition to the rib cage and improving the operating length of the diaphragm due to its cephalad displacement, these improvements in breathing mechanics following LVRS effectively decrease the load on the diaphragm, increase diaphragm pressure-generating capacity, and reduce the level of diaphragm activation needed to support a given level of ventilation (Laghi et al., 1998; Lahrmann et al., 1999; Laghi and Tobin, 2003; Gorman et al., 2005). Therefore, in contrast to AB, enhanced diaphragmatic neuromuscular efficiency following LVRS is due to the combination of increased diaphragm pressure-generating capacity and reduced inspiratory neural drive. Consequently, in the absence of improvements in expiratory flow-generating capacity, static and dynamic breathing mechanics, breathing pattern and EMGdi,rms, isolated and acute improvements in diaphragmatic neuromuscular efficiency during exercise with vs. without AB did not translate into relief of exertional breathlessness and/or improved exercise tolerance in our participants with COPD.

Our findings substantiate the mechanistic role of pathophysiological abnormalities in breathing mechanics and inspiratory neural drive (and deemphasize the mechanistic role of diaphragmatic neuromechanical inefficiency) to the etiology of exertional breathlessness and exercise intolerance in COPD. That is, despite improving pressure-generating capacity and neuromuscular efficiency of the diaphragm, AB had no effect on the inter-relationships between exercise-induced changes in ratings of perceived breathlessness, IRV, breathing pattern and EMGdi,rms. Our findings are consistent with those of Ciavaglia et al. (2014) and Faisal et al. (2016) who, respectively, reported that differences in the activity and recruitment of the diaphragm, accessory inspiratory muscles, and expiratory muscles during walking vs. cycling in obese adults with COPD and during symptom-limited incremental cycle CPET in adults with COPD vs. interstitial lung disease did not influence the relationship between exercise-induced changes in ratings of perceived breathlessness and each of the tidal volume-to-IC ratio (the inverse of IRV), breathing pattern and EMGdi,rms. Collectively, the results add to a growing body of evidence emphasizing the importance of increased inspiratory neural drive in the pathogenesis of exertional breathlessness in COPD (Guenette et al., 2014; Jolley et al., 2015; Elbehairy et al., 2016; Langer et al., 2018; O’Donnell et al., 2018), while simultaneously questioning the role of alterations in the activity of mechanosensitive afferents (i.e., Golgi tendon organs and muscle spindles) emanating from the diaphragm as well as from the chest wall and abdominal muscles in the perception of activity-related breathlessness in COPD.

Compared to CTRL, AB was associated with modest but significant decreases in IC at isotime and peak exercise by ∼110 mL, which may have offset the potentially beneficial effects of enhanced diaphragmatic neuromuscular efficiency on exertional breathlessness and EET. However, this is unlikely, particularly in view of the results of Guenette et al. (2012) who demonstrated that the perception of breathlessness during symptom-limited constant-load CPET in adults with COPD is associated with progressive mechanical constraints on tidal volume expansion as IRV approaches its minimal value, independent of the behavior of dynamic IC. In as much as AB did not affect the behavior of dynamic IRV during exercise, we contend that the small and isolated decreases in IC during exercise with vs. without AB were unlikely to offset the potentially beneficial effects of enhanced diaphragmatic neuromuscular efficiency on exertional breathlessness and EET.

### Methodological Considerations

We evaluated the effects of AB sufficient to increase intra-abdominal pressures by 6.7 ± 0.3 cmH_2_O on the inter-relationships between diaphragmatic neuromuscular efficiency, exertional breathlessness and exercise endurance in adults with COPD. While this level of abdominal compression effectively enhanced diaphragmatic neuromuscular efficiency in the present study as well as in our earlier AB study of healthy younger men (Abdallah et al., 2017), we cannot rule out the possibility that different degrees of abdominal compression may yield different results on diaphragmatic neuromuscular efficiency, exertional breathlessness, and exercise capacity. While the observed changes in diaphragm pressure-generating capacity for a given level of diaphragm electrical activation with AB are consistent with improved length-tension relationship of the diaphragm due to its ascent to a more mechanically advantageous position, we cannot rule out the possibility that cephalad displacement of the diaphragm with AB increased pressure-generating capacity of the diaphragm by decreasing its radius of curvature, even without a change in force generation. Without radiographic evidence of cephalad displacement of the diaphragm with vs. without AB, we can only speculate on the determinants of improved diaphragm pressure-generating capacity and enhanced diaphragmatic neuromuscular efficiency with AB in our participants with COPD. We cannot comment on the effects of AB on cardiac function since measurements of stroke volume and cardiac output were not obtained; however, we have previously demonstrated that AB sufficient to increase intra-abdominal pressures by 6.6 ± 0.6 cmH_2_O had no demonstrable effect on stroke volume and cardiac output responses during constant-load CPET in healthy younger men (Abdallah et al., 2017). As the experimental conditions of this study could not be blinded to the participants and investigators, we cannot rule out the possibility that participant and/or investigator bias may have influenced our results.

## Conclusion

In the absence of improved static and dynamic lung function, expiratory flow-generating capacity, ventilation, breathing pattern, and inspiratory reserve drive, isolated and acute improvements in diaphragmatic neuromuscular efficiency during exercise with AB were not associated with relief of exertional breathlessness and/or improved exercise endurance in adults with COPD.

## Author Contributions

SA, BS, JB, and DJ contributed to the conception of the study, and the collection, analysis and interpretation of data. CW-M contributed to data collection and analysis. PL contributed to data analysis. SA and DJ wrote the manuscript with critical input from all authors. All authors read and approved the final version of the manuscript.

## Conflict of Interest Statement

The authors declare that the research was conducted in the absence of any commercial or financial relationships that could be construed as a potential conflict of interest.
